# Evolution of Neuropsychological Deficits in First-Ever Isolated Ischemic Thalamic Stroke and Their Association With Stroke Topography: A Case-Control Study

**DOI:** 10.1161/STROKEAHA.121.037750

**Published:** 2022-03-09

**Authors:** Anne-Carina Scharf, Janine Gronewold, Olga Todica, Christoph Moenninghoff, Thorsten R. Doeppner, Bianca de Haan, Claudio L.A. Bassetti, Dirk M. Hermann

**Affiliations:** Department of Neurology (A.-C.S., J.G., O.T., D.M.H.), University Hospital Essen, University of Duisburg-Essen, Germany.; Institute of Diagnostic and Interventional Radiology and Neuroradiology (C.M.), University Hospital Essen, University of Duisburg-Essen, Germany.; Department of Neurology, University Medical Center Goettingen, Germany (T.R.D.).; Division of Psychology, Department of Life Sciences, Centre for Cognitive Neuroscience, College of Health, Medicine and Life Sciences, Brunel University London, United Kingdom (B.d.H.).; Department of Neurology, University Hospital Bern, Switzerland (C.L.A.B.).

**Keywords:** brain infarct, cognition, executive function, language, magnetic resonance imaging

## Abstract

The thalamus plays an essential role in cognition. Cognitive deficits have to date mostly been studied retrospectively in chronic thalamic stroke in small cohorts. Studies prospectively evaluating the evolution of cognitive deficits and their association with thalamic stroke topography are lacking. This knowledge is relevant for targeted patient diagnostics and rehabilitation.

**Methods:**

Thirty-seven patients (57.5±17.5 [mean±SD] years, 57% men) with first-ever acute isolated ischemic stroke covering the anterior (n=5), paramedian (n=12), or inferolateral (n=20) thalamus and 37 in-patient controls without stroke with similar vascular risk factors matched for age and sex were prospectively studied. Cognition was evaluated using predefined tests at 1, 6, 12, and 24 months. Voxel-based lesion-symptom mapping was used to determine associations between neuropsychological deficits and stroke topography.

**Results:**

Patients with anterior thalamic stroke revealed severe deficits in verbal memory (median T score [Q1–Q3]: 39.1 [36.1–44.1]), language (31.8 [31.0–43.8]), and executive functions (43.8 [35.5–48.1]) at 1 month compared with controls (verbal memory: 48.5 [43.6–61.0], language: 55.7 [42.3–61.1], executive functions: 51.3 [50.1–56.8]). Patients with paramedian thalamic stroke showed moderate language (44.7 [42.8–55.9]) and executive (49.5 [44.3–55.1]) deficits and no verbal memory deficits (48.1 [42.5–54.7]) at 1 month compared with controls (59.0 [47.0–64.5]; 59.6 [51.1–61.3]; 52.5 [44.2–55.3]). The language and executive deficits in paramedian thalamic stroke patients almost completely recovered during follow-up. Intriguingly, significant deficits in verbal memory (44.7 [41.5–51.9]), language (47.5 [41.8–54.1]), and executive functions (48.2 [46.2–59.7]) were found in inferolateral thalamic stroke patients at 1 month compared with controls (50.5 [46.7–59.9]; 57.0 [51.2–62.9]; 57.4 [51.2–60.7]). Language, but not executive deficits persisted during follow-up. Voxel-based lesion-symptom mapping revealed an association of verbal memory deficits with anterior thalamus lesions and an association of non-verbal memory, language, and executive deficits with lesions at the anterior/paramedian/inferolateral border.

**Conclusions:**

All 3 stroke topographies exhibited significant deficits in diverse cognitive domains, which recovered to a different degree depending on the stroke localization. Our study emphasizes the need for comprehensive neuropsychological diagnostics to secure adequate patient rehabilitation.

The thalamus is connected to a variety of brain regions relevant for cognitive functions via different neuroanatomical circuits.^1^ Lesion studies help us gain insight into thalamic involvement in cognitive processes.^2^ Cognitive deficits in turn are relevant for patient management. Isolated first-ever thalamic strokes are rare, representing about 3% of all ischemic and hemorrhagic strokes.^3^ Few studies prospectively examined cognitive deficits following thalamic stroke by using predefined test protocols and mostly patients were assessed in the chronic stroke phase.^2,4,5^ The majority of studies retrospectively evaluated cognitive deficits in smaller cohorts or case reports.^6–13^

Previous studies suggested that infarcts of the anterior thalamus based on arterial supply^2^ are associated with memory, language, executive, and attention deficits.^2,7,14,15^ In paramedian thalamic brain infarcts, deficits in verbal memory, working memory, language, executive functions, and attention have been reported.^2,10,14,16–19^ In some studies, hemineglect was found.^14,20^ Infarcts of the inferolateral thalamus were associated with information processing slowing.^2,21^ Some studies described language, executive, or memory deficits.^2,14,22^ Because of prominent neurological symptoms like hemiataxia, hemisensory loss, or mild hemiparesis, neuropsychological deficits have frequently not been identified before.^2,4,5^

To date, prospective longitudinal studies comparing neuropsychological performance in patients with thalamic stroke versus control subjects have not been performed. Hence, the temporal evolution of cognitive deficits and its association with thalamic stroke topography remains unknown. This knowledge is however critical for targeted diagnostics and rehabilitation.

## Methods

### Guideline Adherence

This article adheres to the Strengthening the Reporting of Observational Studies in Epidemiology guidelines for reporting case-control studies.

### Data Availability

Additional data can be made available via the corresponding author to qualified researchers upon reasonable request.

### Study Participants

Thirty-seven patients with acute first-ever ischemic thalamic stroke (mean±SD: 57.4±13.0 years, 21 males) and 37 control patients matched for age and sex (57.8±13.5 years) were prospectively recruited at the University Hospital Essen between 2008 and 2017. Patients with stroke were included if they exhibited isolated acute ischemic thalamic stroke at an age ≥18 years. Patients with previous history of psychiatric or neurological disorders, language barriers, severe illnesses, or handicaps precluding neuropsychological examination and follow-up were excluded. Thalamic strokes were diagnosed independently by a neuroradiologist and a neurologist using 1.5-T magnetic resonance imaging (MRI) scans and classified into anterior (n=5), paramedian (n=12), and inferolateral (n=20) infarcts based on arterial supply as suggested before.^2^ Control patients were in-patients matched for age (≤3 years below or above age of respective stroke patient) and sex, with similar vascular risk profile (eg, arterial hypertension, diabetes, hyperlipoproteinemia, overweight, smoking, and history of cardiovascular events in first degree relatives, see Table 1). Control patients were hospitalized in the Department of Neurology for reasons unrelated to acute or chronic CNS pathologies. Control patients did not reveal a previous history for psychiatric or neurological CNS disorders. Patients with stroke and control patients were followed up for 2 years. The study was approved by the Ethics Committee of the University Hospital Essen. All subjects gave written informed consent.

**Table 1.**
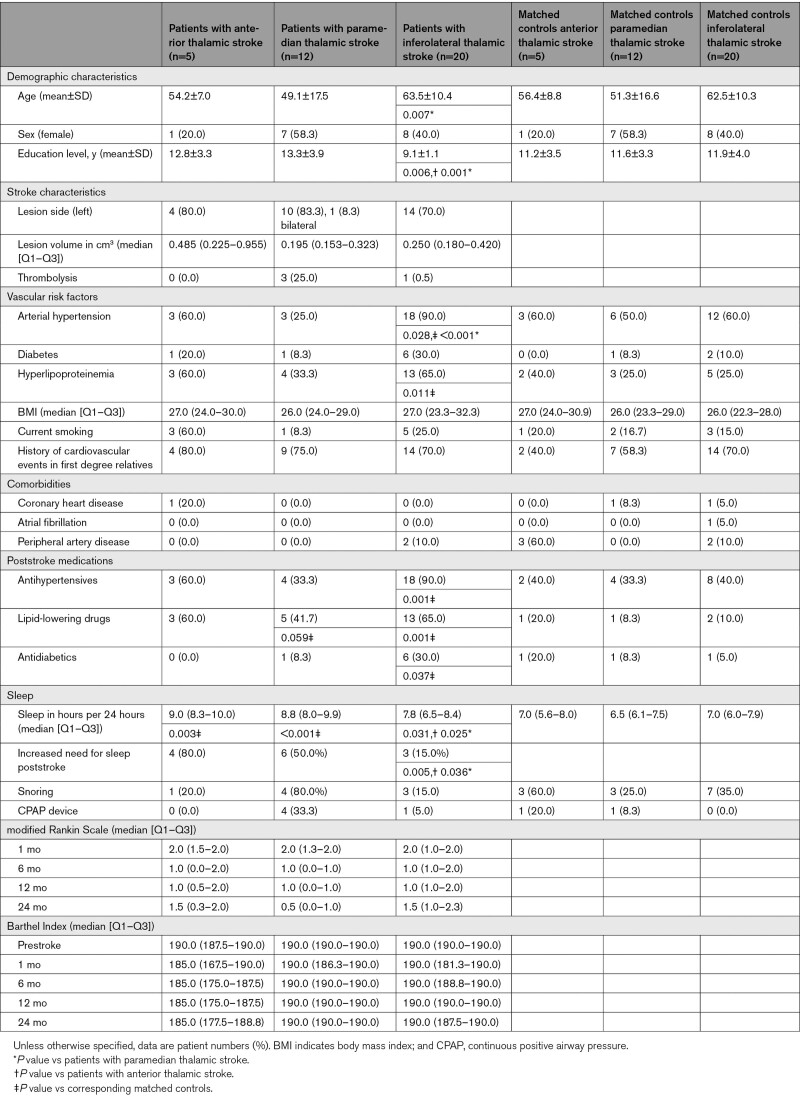
Baseline Characteristics of Patients With Thalamic Stroke and Their Matched Controls

Of 45 patients with first-ever acute ischemic thalamic stroke, 1 patient each was excluded because of schizophrenia, language barriers, or severe illness. 5 patients refused participation. Thus, 37 patients were enrolled, of whom all could be followed up for 12 months and 29 for 24 months poststroke (Figure S1). In 2 patients (1 with anterior thalamic stroke, 1 with inferolateral thalamic stroke), MRI scans could not be performed due to severe claustrophobia.

### Medical History, Clinical and Laboratory Tests

Upon admission (median [Q1–Q3]=6.8 [1.0–24.0] hours poststroke), a detailed patient interview was performed to collect sociodemographic data and a medical history, followed by a physical examination, laboratory, blood, and urine tests. Arterial hypertension was defined as systolic blood pressure ≥140 mmHg, diastolic blood pressure ≥90 mm Hg, or antihypertensive drug prescription, diabetes as physician–diagnosed diabetes, blood glucose >200 mg/dL, fasting glucose >126 mg/dL, or antidiabetic medication prescription, nicotine abuse as current smoking. Standardized height and weight measurements were performed to calculate the body mass index. Education level was assessed as years of academic education in school and university. Sleep was evaluated by assessing the current (poststroke) number of hours sleep/24 hours, need to sleep poststroke (categorized as increased or unchanged as compared with before stroke), history of snoring (yes/no), and continuous positive airway pressure use (yes/no).

### Neurological and Neuropsychological Assessment

Upon admission, a detailed neurological examination was performed by a staff neurologist. Following study inclusion after written informed consent, detailed neurological and neuropsychological examinations were performed by a neurologist and neuropsychologist at 1 (1.1 [1.0–1.4]), 6 (5.5 [5.0–6.0]), 12 (12.0 [11.0–12.0]) and 24 (24.0 [23.0–24.25]) months. On each visit, the extended Barthel Index and the modified Rankin Scale score were determined to assess activities of daily living and the degree of disability.

The neuropsychological assessment (for further details, see Table S1) included the evaluation of

1. Verbal memory via digit span forwards and backwards (number of correctly recalled sequences)^23^ and Rivermead behavioral memory test—story immediate and delayed recall (number of correctly recalled items),^24^
2. Non-verbal memory via block span forward and backward,^23^
3. Language via the Regensburg semantic and phonemic word fluency test (subtests animals, food, occupation or hobby, and s-words, p-words, m-words or k-words administered sequentially to prevent retest effects; number of correctly called words),^25^
4. Executive functions via the trail making test parts A (assessment of information processing speed; time needed to perform the test) and B (assessment of cognitive flexibility; time needed to perform the test),^26^ and the Stroop color word test (assessment of cognitive interference; interference score),^27^ and
5. Attention via the test of attentional performance alertness (median reaction time with and without warning tone), Go/No-go (median reaction time), and divided attention (median visual and auditive reaction time) tests.^28^

T scores were calculated based on reference values published in test manuals (see Table S1).

### MRI Data Acquisition and Preprocessing

Detailed structural MRI examinations including T1-, T2-, fluid attenuated-, diffusion-weighted, and susceptibility-weighted sequences were performed at 1 month, 6 months and 24 months poststroke on a 1.5 T scanner (MagnetomAvanto; Siemens Healthcare, Erlangen, Germany) with a standard 8-channel birdcage head coil. For voxel-based lesion-symptom mapping (VLSM), the T1-weighted volumetric magnetization prepared rapid acquisition gradient echo (MP-RAGE) sequence was used (TR/TE/TI=2400/3.52/1200 ms, flip angle=8°, 256×265 mm^2^ matrix, 1.0×1.0×1.0 mm^3^ resolution, 160 slices). Preprocessing was performed using MRIcroN^29^ and Statistical Parametric Mapping version 12 (SPM12) software implemented in Matlab R2016a (MathWorks, Natick, MA). As automated lesion drawing was not reliable in detecting small thalamic lesions, MRIcroN was used to manually label lesion boundaries in each patient. MRI scans and lesion maps were transformed into stereotactic space using SPM12 and the Clinical Toolbox, which includes a normalization template for aged brains. To determine the transformation parameters, cost-function masking was used.^30,31^

### Statistical Analyses

Results were presented as median (Q1–Q3) for continuous data because of non-normal distribution and as numbers (%) for categorical data. Clinical characteristics and neuropsychological data were compared (1) between anterior, inferolateral and paramedian thalamic stroke patients and their corresponding matched controls and (2) among each other between anterior, inferolateral, and paramedian thalamic stroke patients using Mann-Whitney *U* tests for continuous data, and Pearson χ^2^ tests or Fisher exact tests for categorical data. Longitudinal comparisons were performed with Wilcoxon signed-rank test for continuous data and McNemar test for categorical data.

Using the normalized lesion maps, VLSM was performed to determine the statistical association between voxel status (lesioned or intact) and neuropsychological test scores.^32,33^ Since the neuropsychological data were continuous, Brunner-Munzel tests were performed using nonparametric mapping software.^34,35^ To ensure statistical power, voxels exhibiting a lesion in <5 patients were excluded from analysis. To correct for multiple comparisons, a false discovery rate correction was used. *P* ≤0.050 were considered statistically significant. Missing data were excluded listwise. Statistical analyses were performed with IBM SPSS Statistics for Windows, Version 22.0. Armonk, NY: IBM Corp. For anatomic interpretation of the results, the statistical maps were registered onto axial slices of Morel’s stereotactic atlas.^36^

## Results

### Demographic and Clinical Characteristics

Demographic and clinical patient characteristics are provided in Table 1. Of 37 stroke patients, 27 (71.7%) had a unilateral left, 9 (24.3%) had a unilateral right, and 1 (2.6%) had a bilateral stroke. Five (13.5%) patients had anterior, 12 (32.4%) paramedian, and 20 (54.1%) inferolateral thalamic stroke. Patients with anterior, paramedian, and inferolateral thalamic stroke did not significantly differ in lesion volume. Patients with inferolateral thalamic stroke were older than patients with paramedian thalamic stroke, less educated than patients with anterior and paramedian thalamic stroke, and more often suffered from vascular risk factors. Poststroke sleep needs were increased in patients with anterior and paramedian thalamic stroke when compared with control patients. Notably‚ all latter stroke patients reported an increase of sleep needs as compared with prestroke values. Disability recovered best in patients with paramedian thalamic stroke. Patients with paramedian and inferolateral thalamic stroke reached functional independence at 6 months.

### Neurological Examination

The detailed time course of neurological deficits is given in Table S2. Patients with anterior thalamic stroke mostly revealed aphasia, pupillomotor deficits, sensory deficits affecting the face, gait ataxia, sensory deficits affecting the extremities and mild hemiparesis upon admission. Whereas aphasia, pupillomotor deficits, sensory deficits, and hemiparesis recovered during follow-up, mild gait ataxia persisted. Patients with paramedian thalamic stroke presented with aphasia, pupillomotor deficits, facial sensory deficits, facial hemiparesis, gait ataxia, limb ataxia, extremital sensory deficits, and mild hemiparesis upon admission. These deficits almost fully recovered during follow-up. Inferolateral thalamic strokes revealed aphasia, dysosmia, pupillomotor deficits, facial sensory deficits, facial hemiparesis, dysphagia, gait ataxia, limb ataxia, sensory deficits of the extremities, and mild hemiparesis upon admission. These deficits only partly recovered during follow-up.

### Neuropsychological Assessment

**Figure 1.**
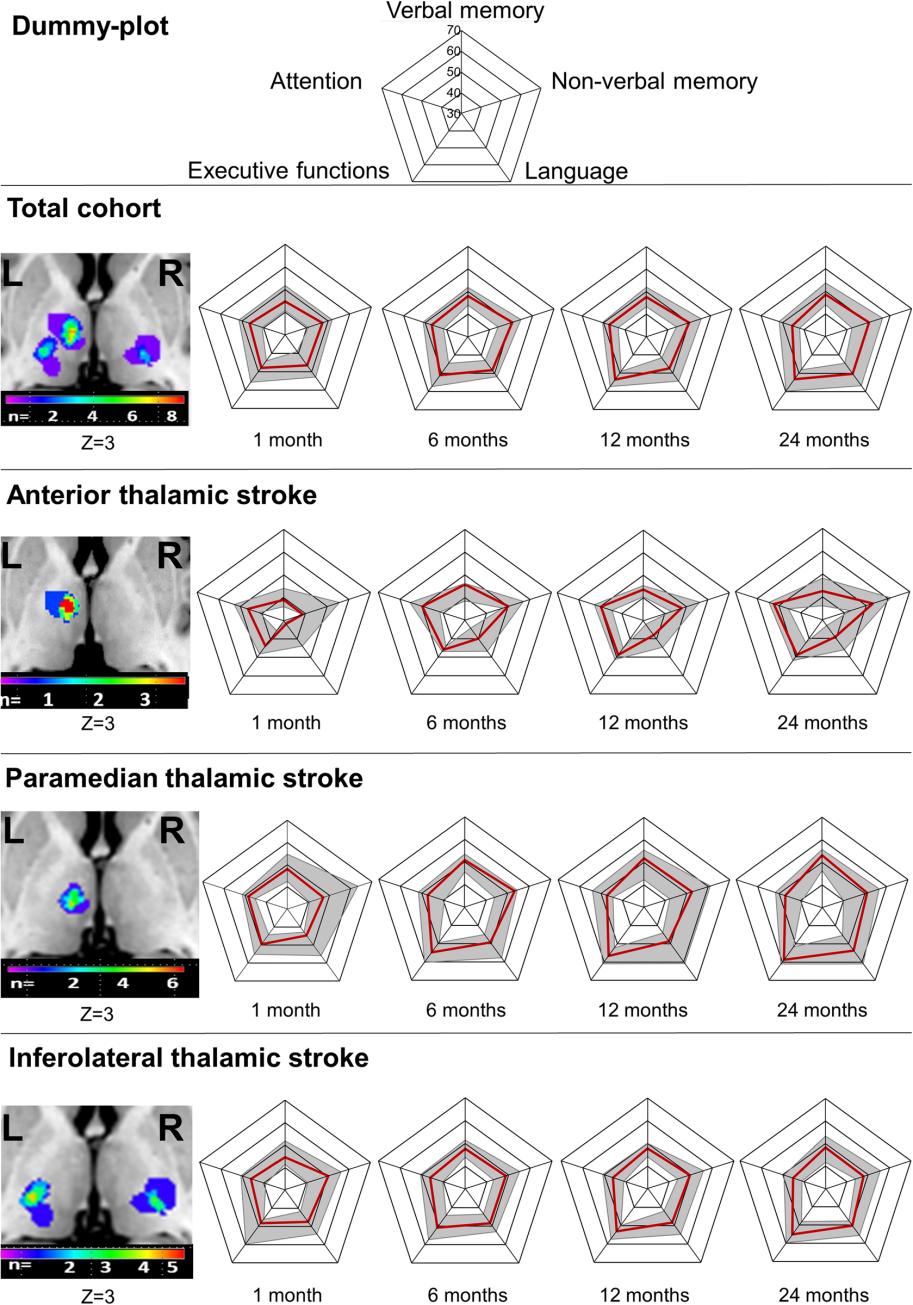
**T scores of different cognitive domains for all strokes and strokes split by vascular territory at 1, 6, 12, and 24 mo poststroke.** Cognitive data are median (solid line)±interquartile ranges (shaded areas). On the left side, lesion overlay maps are shown, for further details see Figure 2. Single infarcts were located outside this plane.

Because of vigilance disturbances, sleepiness and behavioral abnormalities including restlessness in the acute stroke phase, neuropsychological performance was not systematically evaluated upon admission but first examined after 1 month. A graphical longitudinal overview of neuropsychological performance for the cognitive domains from 1 month to 24 months poststroke is presented in Figure 1, the detailed time course for the cognitive domains is given in numbers in Table 2, T scores and raw scores of individual neuropsychological tests are shown in Tables S3 and S4, respectively.

**Table 2.**
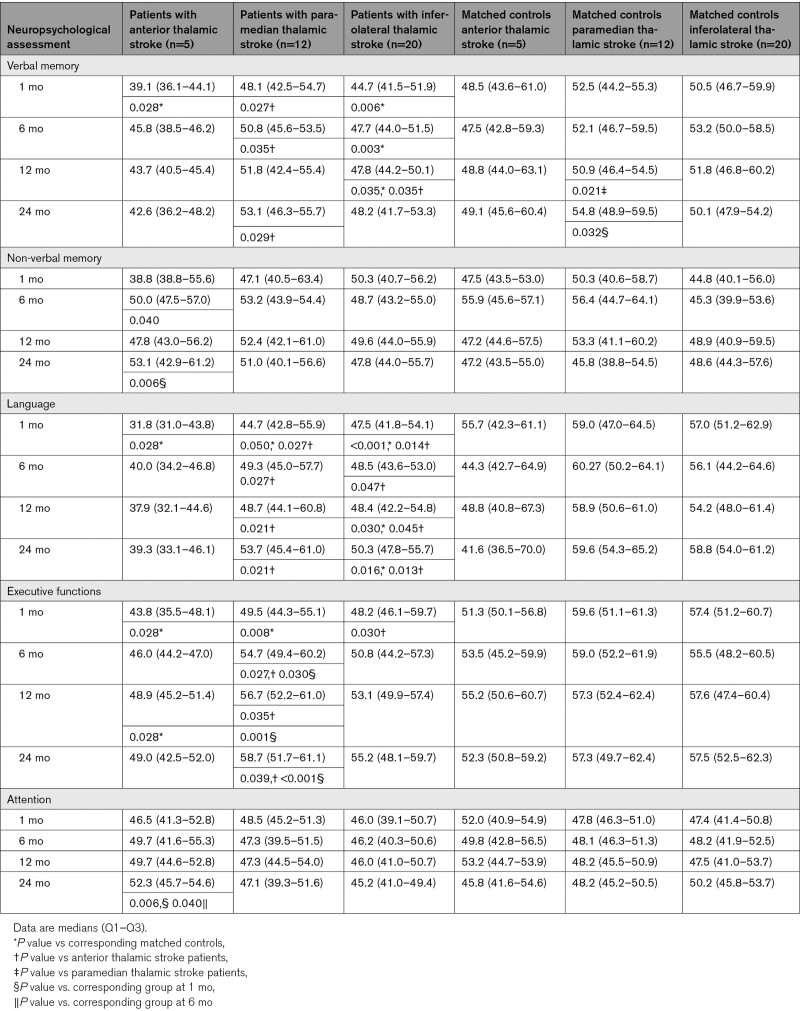
T Scores of Cognitive Domains in Patients With Thalamic Stroke and Their Matched Controls

#### Verbal Memory

Patients with anterior thalamic stroke showed particularly severe verbal memory deficits at 1 month compared with their matched controls. These deficits slightly improved during follow-up. Patients with paramedian thalamic stroke had no significant deficits in verbal memory compared with their matched controls. Their verbal memory performance was significantly better than that of patients with anterior thalamic stroke. Inferolateral thalamic stroke patients had significant verbal memory deficits at 1 month compared with their matched controls. Verbal memory largely recovered to the level of matched controls.

#### Non-Verbal Memory

Patients with anterior thalamic stroke showed weak non-verbal memory performance at 1 month compared with their matched controls. Non-verbal memory of anterior thalamic stroke patients improved during follow-up. Non-verbal memory of paramedian and inferior thalamic stroke patients did not significantly differ from matched controls.

#### Language

Severe language deficits were noted in patients with anterior thalamic stroke at 1 month compared with their matched controls. Language deficits improved with largely no residual language deficits being observed after 24 months. Likewise, significant language deficits were found in patients with paramedian thalamic stroke at 1 month compared with their matched controls. These language deficits largely recovered during follow-up. Significant language deficits were also detected in patients with inferolateral thalamic stroke at 1 month compared with their matched controls. These deficits largely persisted at 24 months.

#### Executive Functions

In patients with anterior thalamic stroke, significant impairments of executive functions were found at 1 month compared with their matched controls. Executive functions largely recovered during follow-up. Likewise, significant deficits of executive functions were found in patients with paramedian thalamic stroke at 1 month compared with their matched controls. These deficits fully recovered during follow-up.

When looking at individual neuropsychological tests, especially trail making test part B, assessing cognitive flexibility‚ was impaired in anterior and paramedian thalamic stroke patients (Tables S3 and S4). Executive deficits were also noted in patients with inferolateral thalamic stroke at 1 month compared with their matched controls. These deficits fully recovered during follow-up.

#### Attention

Attention neither differed between patients with stroke and their matched controls nor between stroke topographies.

### Analysis of Structure-Function Relationship Using VLSM

**Figure 2.**
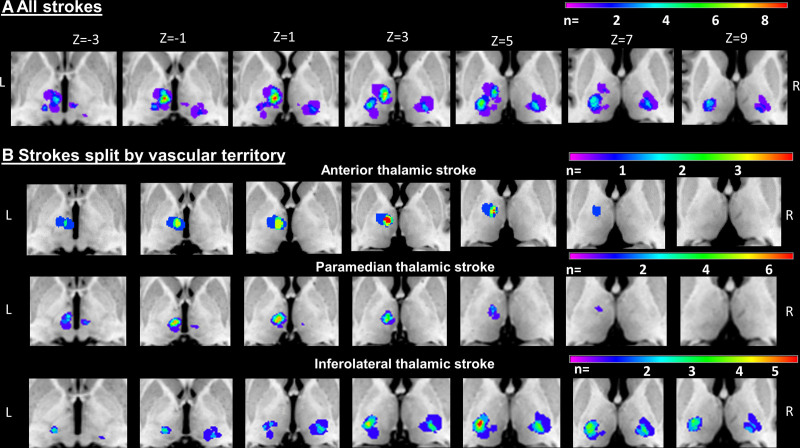
**Lesion overlay map showing the anterior, paramedian, and inferolateral thalamic lesions.** The color code indicates in how many patients of the cohort a given voxel was lesioned. Z coordinates relate to MNI space. **A**, All strokes, (**B**) strokes split by vascular territory. Magnetic resonance images are in neurological view (left side represents left hemisphere).

The lesion overlap map of all stroke patients showed that the thalamic regions most often affected were in the left central thalamus (X=−8, Y=−14, Z=1) with 9 patients showing overlapping lesions (Figure 2). In the left (X=−19, Y=−19, Z=7) and right (X=20, Y=−21, Z=3) inferolateral thalamus 5 patients each showed overlapping lesions.

#### Verbal Memory

**Figure 3.**
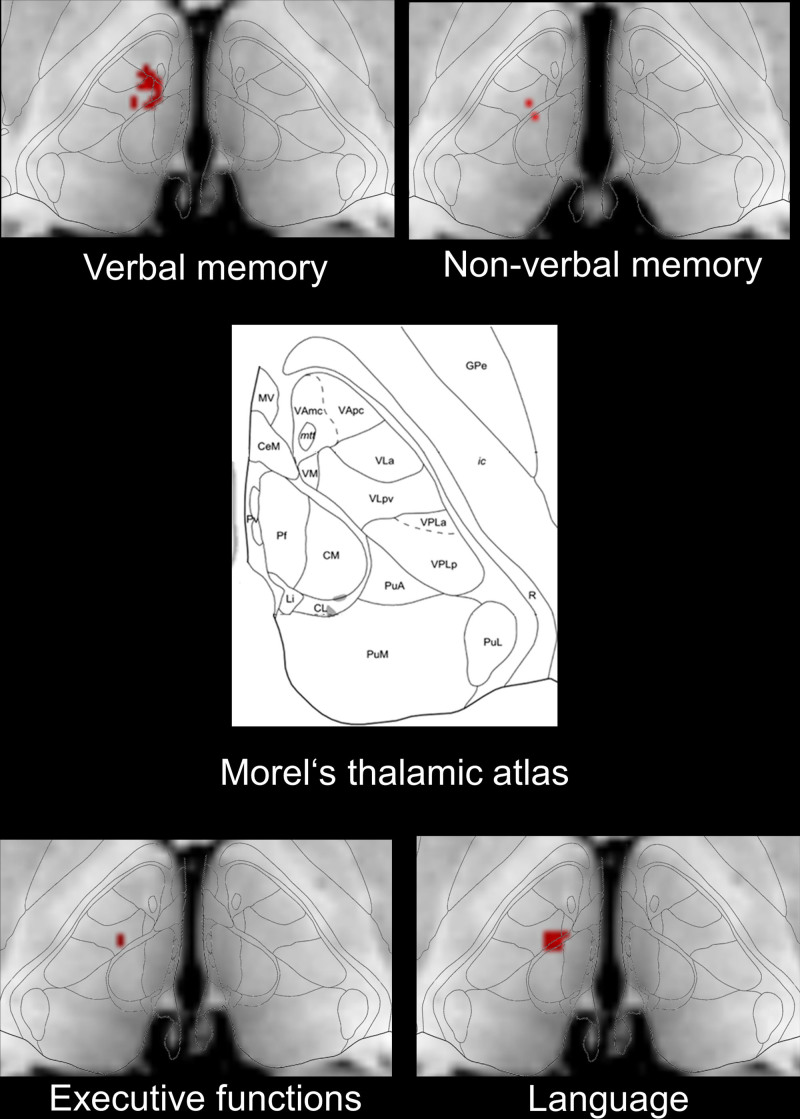
**Voxel-based lesion-symptom mapping of neuropsychological deficits at 1 mo.** Data are shown in neurological view (left side is left hemisphere, Z-level=2). Cd indicates caudate nucleus; CeM, central medial nucleus; CL, central lateral nucleus; CM, central median nucleus; GPe, globus pallidum external; Hb, habenula; ic, internal capsule; Li, limitans nucleus; mtt, mammillothalamic tract; MV, medioventral nucleus; Pf, parafascicular nucleus; PuA, anterior pulvinar nuclei; PuL, lateral pulvinar nuclei; PuM, medial pulvinar nuclei; Pv, paraventricular nucleus; PuT, putamen; R, reticular nucleus; VA, ventral anterior nucleus; VAmc, ventral anterior nucleus magnocellular division; VApc, ventral anterior nucleus parvocellular division; VLa, ventral lateral anterior nucleus; VM, ventral medial nucleus; VLpv, ventral posterior lateral nucleus, parvocellular division; VPLa, ventral posterior lateral nucleus, anterior division; and VPLp, ventral posterior lateral nucleus, posterior division.

VLSM confirmed the association of damaged voxels in the left anterior thalamus and deficits in verbal memory (Figure 3). Significant voxels were located around the MNI coordinates X=−9, Y=−11, and Z=2 (*z*max=3.06, *P*<0.001; *z*FDR-corrected=2.98, *P*=0.002), which corresponded to the ventral anterior thalamic nuclei, mammillothalamic tract, ventral lateral thalamic, and ventral medial thalamic nuclei.^36^

#### Non-Verbal Memory

VLSM also confirmed the association of damaged voxels at the border between the left anterior and paramedian thalamus and non-verbal memory deficits. Damage to voxels around MNI coordinates X=−11, Y=−14, and Z=1 significantly predicted the severity of non-verbal memory deficits (*z*max=2.11, *P*=0.030). These voxels corresponded to the central medial and ventral lateral thalamic nuclei.

#### Language

Damage to voxels around MNI coordinates X=−9, Y=−14, and Z=2 in the left anterocentral thalamus significantly predicted the severity of language deficits (*z*max=3.19, *P*=0.001; *z*FDR-corrected=2.98, *P*=0.001). These MNI coordinates represented the central medial, ventral medial, and ventral lateral thalamic nuclei.

#### Executive Functions

Damage to voxels around MNI coordinates X=−11, Y=−13, and Z=2 significantly predicted the severity of executive function deficits (*z*max=3.19, *P*=0.001; *z*FDR-corrected=3.66, *P*<0.001). These MNI coordinates represented the ventral lateral thalamic nucleus.

#### Attention

VLSM could not identify any significant associations between voxel lesion status and attention deficits.

## Discussion

### Key Findings

In our prospective longitudinal study, we for the first time analyzed the temporal evolution of cognitive deficits in thalamic strokes affecting 3 different vascular territories. Our main findings were (1) patients with anterior thalamic stroke had the most severe deficits in verbal memory, language, and executive functions, which poorly recovered during follow-up; (2) patients with paramedian thalamic stroke revealed more moderate language and executive deficits, which recovered best among the 3 stroke topographies almost to the level of control patients; (3) inferolateral stroke patients also suffered from verbal memory, language and executive deficits; the language deficits persisted, whereas the verbal memory and executive deficits recovered during follow-up; and (4) VLSM confirmed an association of verbal memory deficits with lesions of the left anterior thalamus and an association of non-verbal memory, language, and executive deficits with lesions at the border between the left anterior, paramedian and inferolateral thalamus.

### Comparison With Previous Evidence

In one of the few prospective studies, Ghika-Schmidt and Bogousslavsky in line with our study reported memory and executive function deficits in 12 anterior thalamic stroke patients within 5 days poststroke.^7^ On the behavioral level, confusional states associated with perseveration and confabulation were found. Unlike in our study, neuropsychological recovery was not systematically examined in follow-up examinations and control subjects were not included. VLSM specifically showed an association between verbal memory deficits and lesions of the left anterior thalamus in our study. Thus, our results in line with previous research findings support a central role of the anterior thalamus for memory formation and recollection.^6^ Lesions associated with verbal memory deficits in our study included the mammillothalamic tract, which, as part of the medial limbic circuit, connects the anterior thalamic nuclei with the hippocampus.^6,9^ Mammillothalamic tract lesions have previously been shown to be associated with pronounced memory disturbances.^10,37,38^

Previous studies in paramedian stroke patients yielded mixed results. Some studies contrary to our study specifically observed memory deficits^9,13,14,39^ whereas other studies, like our study, showed a broader range of cognitive deficits.^2,4,5,19^ Some studies analyzed cognitive deficits during the first days poststroke,^14^ while other studies analyzed cognitive deficits in the chronic phase 1 to 5 years poststroke.^13,19,39^ Previous studies revealing memory deficits included a higher number of bilateral paramedian strokes,^14,39^ while our sample only included 1 patient with bilateral paramedian stroke. Similar to our study, a previous study analyzing 19 mostly chronic thalamic stroke patients (0.25–120 months poststroke) by subtraction analysis showed that in 8 patients with executive deficits mainly an area in the left medial thalamus consisting of the centromedian and parafascicular nuclei was damaged.^19^ Using VLSM, we identified regions at the border between the left anterior, paramedian, and inferolateral thalamus that predicted non-verbal memory, language, and executive impairment. Due to defined time points and the larger sample size, our study was better powered to identify associations between neuropsychological deficits and stroke topography. Interestingly, the mediodorsal thalamic nucleus was not associated with memory or executive deficits in our study. The mediodorsal thalamic nucleus seems to play a multifaceted role in higher cognitive functions and mediate cortical network regulation.^9,38^

Because of prominent neurological symptoms, cognitive deficits following inferolateral thalamic stroke were frequently overlooked in earlier studies.^4,5^ More recently, infarcts of the inferolateral thalamus were shown to be associated with slowing of information processing speed.^2,21^ In line with our results, a study evaluating 9 patients with inferolateral thalamic stroke with neuropsychological assessments performed 3 to 6 months poststroke, observed memory, language, and executive deficits as main neuropsychological symptoms.^22^ Using bedside assessments, language disturbances, but no memory or executive deficits were noted in the first week poststroke in 30 patients with ischemic inferolateral thalamic stroke.^11^ In the study including thalamic stroke patients mostly in the chronic phase, memory, language, and executive deficits could not be detected in patients with stroke affecting the inferolateral thalamic region.^19^

In our study, VLSM revealed a region at the border between the anterior, paramedian, and inferolateral thalamus in which damage predicted non-verbal memory, language, and executive impairments. Interestingly, the region associated with executive deficits was slightly more lateral (X=−11, Y=−13, Z=2) than the region associated with language deficits (X=−9, Y=−14, Z=2). The region located at X=−11, Y=−13, Z=2 represented the ventral lateral thalamic nuclei, supporting the hypothesis that anatomic circuits between these nuclei and the cerebral cortex are involved in executive tasks.^22^

### Strengths and Limitations

To date, most studies retrospectively assessed cognitive deficits in smaller cohorts or case reports, and the majority of them used chronic stroke patients without matched control subjects.^6–13,40^ Longitudinal studies comparing neuropsychological deficits of patients with thalamic stroke with age- and sex-matched control subjects at predefined time points with a standardized comprehensive neuropsychological test battery were lacking. Whereas our longitudinal design with 24 months follow-up allows for the assessment of long-term stroke recovery, we were able to track the complete cohort of 37 patients only for 12 months. A total of 8 patients were missing at the 24-month follow-up (5 refused participation, 2 had reinfarcts, 1 died). This could lead to systematic bias in the patient evaluation at the 24-month time point. The 8 patients lost to follow-up, however, did not statistically differ in age, sex, and each of the cognitive domains before their dropout from patients who completed the 24-month follow-up. We did not perform any systematic assessment of prestroke cognitive performance in patients with thalamic stroke. Yet, in the detailed patient interview upon admission, neither the patients nor accompanying relatives nor previous medical records reported prestroke cognitive deficits. The 3 thalamic stroke topographies evaluated in our study had different age, sex, and vascular risk profiles: patients with paramedian thalamic stroke were younger, more often female, and healthier than anterior and inferolateral thalamic stroke patients, which exemplifies the importance of the right selection of control subjects for evaluating stroke-associated cognitive deficits. Because of the predominance of left-hemispheric stroke, non-verbal memory was not affected to a major extent in our sample. The predominance of left hemispheric strokes is, however, a typical finding in thalamic stroke studies.^7^ Using MRI, we evaluated the association of neuropsychological results with stroke topography in 2 different ways, that is, by arterial supply and VLSM, which assesses the association of clinical symptoms and stroke lesions without anatomic preassumptions. Although our study is the first study, which used VLSM to study cognitive deficits in patients with acute thalamic stroke, it has to be noted that the statistical power of observations depends on sample size. In the present study, only 5 of 37 patients with thalamic stroke had an anterior thalamic stroke, which is in line with prevalence rates in previous studies.^2^ Importantly, cognitive deficits were particularly severe in patients with anterior thalamic stroke, which allowed us to reliably delineate the neuropsychological consequences associated with this stroke topography.

### Clinical Implications

Our study offers valuable information for patient diagnostics and rehabilitation. Especially in patients with inferolateral thalamic stroke, neuropsychological deficits are often not recognized because these deficits are clouded by the more prominent focal neurological symptoms. Poststroke cognitive impairment is a key driver of poor stroke outcome, impairing activities of daily living and augmenting the costs of health care,^41^ decreasing the quality of life^42^ and increasing the institutionalization rate.^43^ Meanwhile, various cognitive rehabilitation treatments have been shown to improve stroke recovery,^44^ although continued uncertainty persists regarding the benefits and limitations of several interventions.^45^ The negligence of cognitive deficits excludes these patients from appropriate rehabilitation measures. With respect to the evolution of cognitive deficits, our study offers useful prognostic information, which may help to predict stroke outcome and thus to estimate the future need of care.

## Article Information

### Sources of Funding

None.

### Disclosures

None.

## Supplementary Material


