# Supplementary material for: Evolution of Neuropsychological Deficits in First-Ever Isolated Ischemic Thalamic Stroke and Their Association With Stroke Topography: A Case-Control Study
Source: Stroke. 2022 Mar 9;53(6):1904–14. doi: 10.1161/STROKEAHA.121.037750 (PMC9126267; doi:10.1161/STROKEAHA.121.037750)
Supplement: Supplementary file 1 [file str-53-1904-s001.pdf]

SUPPLEMENTAL MATERIAL

Evolution of neuropsychological deficits in first-ever isolated ischemic thalamic stroke and their association with stroke topography – a case-control study

Anne-Carina Scharf, MSc[1] Janine Gronewold, PhD[1] Olga Todica, MSc[1] Christoph Moenninghoff, MD[2] Thorsten R. Doeppner, MD, MSc[3] Bianca de Haan, PhD[4] Claudio L. Bassetti, MD[5] Dirk M. Hermann, MD[1]

[1]Department of Neurology and [2]Institute of Diagnostic and Interventional Radiology and Neuroradiology, University Hospital Essen, University of Duisburg-Essen, Essen, Germany; [3]Department of Neurology, University Medical Center Goettingen, Goettingen, Germany; [4]Division of Psychology, Department of Life Sciences, Centre for Cognitive Neuroscience, College of Health, Medicine and Life Sciences, Brunel University London, U.K.; [5]Department of Neurology, University Hospital Bern, Bern, Switzerland

Number of supplementary figures: 1
Number of supplementary tables: 4

Table of content

Figure S1 Thalamic stroke patient recruitment
Table S1 Comprehensive neuropsychological assessment
Table S2 Neurological deficits in thalamic stroke patients
Table S3 T scores of individual neuropsychological tests in thalamic stroke patients and their matched controls
Table S4 Raw data of individual neuropsychological tests in thalamic stroke patients and their matched controls

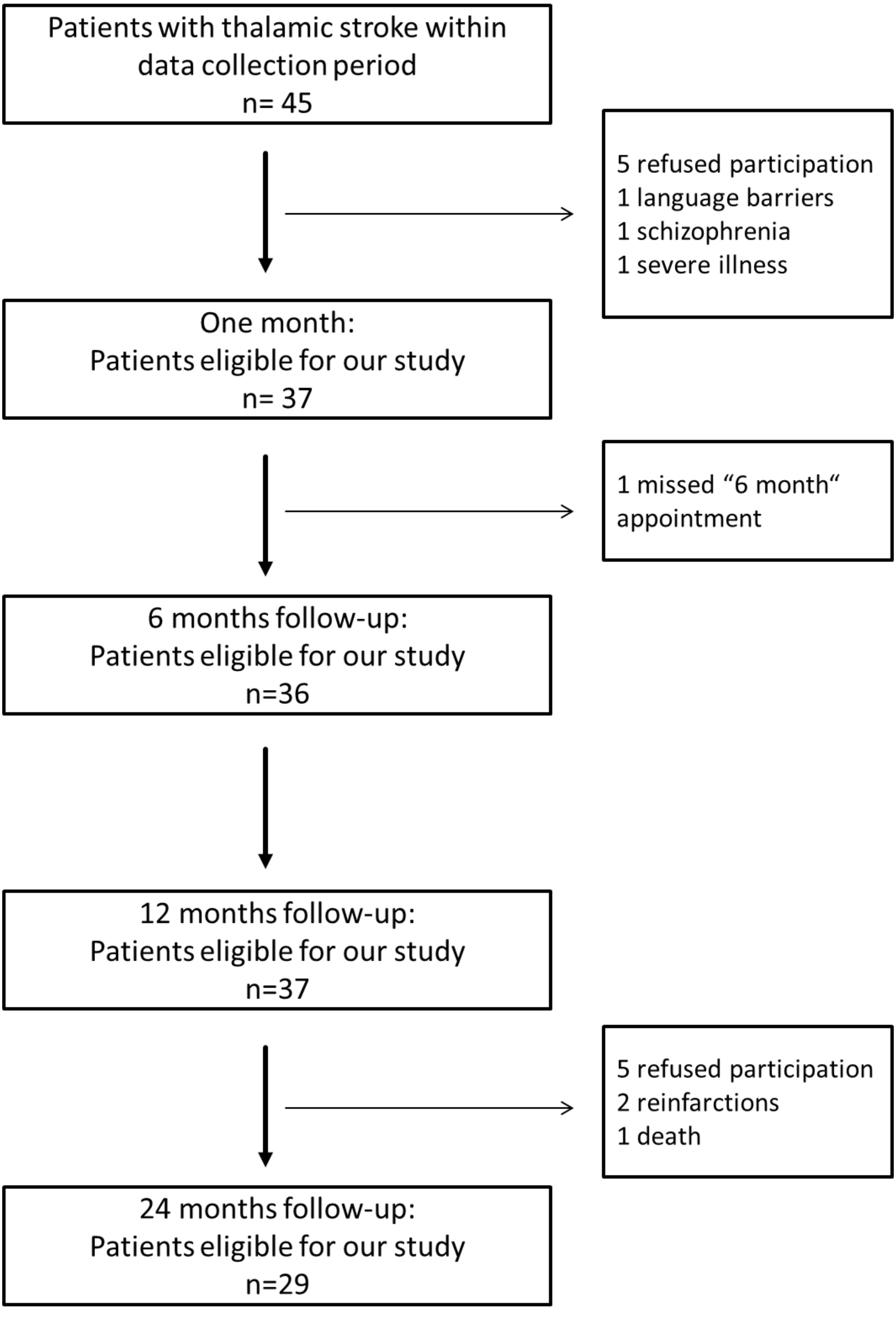

Figure S1. Thalamic stroke patient recruitment. Patients included and excluded at various points of examination are shown.

Table S1. Comprehensive neuropsychological assessment

Cognitive domain	Neuropsychological tests	Subtests	References
Verbal memory	Digit span forwards	Number of correctly recalled items	Härting et al. Wechsler Gedächtnis Test-Revidierte Fassung (WMS-R). Bern: Huber. 2000
	Digit span backwards	Number of correctly recalled items	
	Rivermead behavioral memory test (RBMT)	Number of correctly recalled items	Wilson et al. Rivermead Behavioural Memory Test (RBMT)–Deutsche Version. 1992
Non-verbal memory	Block span forwards	Number of correctly recalled items	Härting et al. Wechsler Gedächtnis Test-Revidierte Fassung (WMS-R). Bern: Huber. 2000
	Block span backwards	Number of correctly recalled items	
Language	Regensburg semantic and phonemic word fluency test	'animals', 'food', 'occupation' or 'hobby', plus 's-words', 'p-words', 'm-words' or 'k-words'	Aschenbrenner et al. Regensburger Wortflüssigkeitstest. Göttingen: Hogrefe. 2000
Executive functions	Trail making test (TMT) part A	Assessment of information processing speed; time needed to perform the test	Reitan RM & Wolfson D. The Halstead-Reitan Neuropsychological Test Battery: Theory and Clinical Interpretation. Tucson, AZ: Neuropsychology Press. 1985. Tombaugh TN. Trail making test a and b: Normative data stratified by age and education. Archives of Clinical Neuropsychology. 2004;19:203-214
	Trail making test (TMT) part B	Assessment of cognitive flexibility; time needed to perform the test	
	Stroop color word test	Assessment of cognitive interference; interference score	Stroop JR. Studies of interference in serial verbal reactions. Journal of experimental psychology. 1935;18:643
Attention	Test of Attentional Performance (TAP) Alertness	median reaction time with and without warning tone	Zimmermann P, Fimm B. A test battery for attentional performance. In: Leclercq M, Zimmermann P, eds. Applied neuropsychology of attention: Theory, diagnosis and rehabilitation. New York: Psychology Press; 2002:110-151.
	Test of Attentional Performance (TAP) Go/No-go	median reaction time	
	Test of Attentional Performance (TAP) Divided attention	median visual and auditive reaction time	

Table S2. Neurological deficits in thalamic stroke patients

Deficit in n(%)	Anterior thalamic stroke patients					Paramedian thalamic stroke patients					Inferolateral thalamic stroke patients				
	Acute phase (n=5)	*1 month (n=5)*	*6 months (n=5)*	*12 months (n=5)*	*24 months (n=4)*	*Acute phase (n=12)*	*1 month (n=12)*	*6 months (n=12)*	*12 months (n=12)*	*24 months (n=12)*	*Acute phase (n=20)*	*1 month (n=20)*	*6 months (n=19)*	*12 months (n=20)*	*24 months (n=13)*
Aphasia	1 (20.0)	0 (0.0)	0 (0.0)	0 (0.0)	0 (0.0)	6 (50.0)	1 (8.3)	1 (8.3)	1 (8.3)	0 (0.0)	5 (25.0)	1 (5.0)	1 (5.2)	1 (5.0)	0 (0.0)
Dysosmia	1 (20.0)	1 (20.0)	1 (20.0)	1 (20.0)	1 (25.0)	1 (8.3)	1 (8.3)	1 (8.3)	1 (8.3)	1 (8.3)	15 (75.0)	13 (65.0)	12 (63.2)	12 (60.0)	7 (53.8)
Visual deficits	1 (20.0)	0 (0.0)	0 (0.0)	0 (0.0)	0 (0.0)	0 (0.0)	0 (0.0)	0 (0.0)	0 (0.0)	0 (0.0)	0 (0.0)	0 (0.0)	0 (0.0)	0 (0.0)	0 (0.0)
Oculomotor deficits	0 (0.0)	0 (0.0)	0 (0.0)	0 (0.0)	0 (0.0)	0 (0.0)	0 (0.0)	0 (0.0)	0 (0.0)	0 (0.0)	0 (0.0)	0 (0.0)	0 (0.0)	0 (0.0)	0 (0.0)
Pupillomotor deficits	1 (20.0)	0 (0.0)	0 (0.0)	0 (0.0)	0 (0.0)	2 (16.7)	1 (8.3)	0 (0.0)	0 (0.0)	0 (0.0)	2 (10.0)	3 (15.0)	2 (10.5)	1 (5.0)	0 (0.0)
Facial sensory deficits	2 (40.0)	1 (20.0)	0 (0.0)	0 (0.0)	0 (0.0)	6 (50.0)	1 (8.3)	0 (0.0)	0 (0.0)	0 (0.0)	6 (30.0)	1 (5.0)	0 (0.0)	1 (5.0)	1 (7.7)
Facial hemiparesis	0 (0.0)	0 (0.0)	0 (0.0)	0 (0.0)	0 (0.0)	3 (25.0)	1 (8.3)	1 (8.3)	0 (0.0)	0 (0.0)	1 (5.0)	0 (0.0)	0 (0.0)	0 (0.0)	0 (0.0)
Dysgeusia	0 (0.0)	0 (0.0)	0 (0.0)	0 (0.0)	0 (0.0)	0 (0.0)	0 (0.0)	0 (0.0)	0 (0.0)	0 (0.0)	0 (0.0)	0 (0.0)	1 (5.2)	1 (5.0)	0 (0.0)
Dysphagia	0 (0.0)	0 (0.0)	0 (0.0)	0 (0.0)	0 (0.0)	0 (0.0)	0 (0.0)	0 (0.0)	0 (0.0)	0 (0.0)	11 (55.0)	6 (30.0)	3 (15.8)	4 (20.0)	2 (15.4)
Dysarthria	0 (0.0)	0 (0.0)	0 (0.0)	0 (0.0)	0 (0.0)	0 (0.0)	0 (0.0)	0 (0.0)	0 (0.0)	0 (0.0)	0 (0.0)	1 (5.0)	1 (5.2)	1 (5.0)	1 (7.7)
Gait ataxia	3 (60.0)	4 (80.0)	4 (80.0)	2 (40.0)	2 (50.0)	4 (33.3)	3 (25.0)	1 (8.3)	1 (8.3)	1 (8.3)	1 (5.0)	1 (5.0)	2 (10.5)	1 (5.0)	0 (0.0)
Limb ataxia	0 (0.0)	0 (0.0)	0 (0.0)	0 (0.0)	0 (0.0)	4 (33.3)	2 (16.7)	0 (0.0)	0 (0.0)	1 (8.3)	10 (50.0)	9 (45.0)	3 (15.8)	3 (15.0)	3 (23.1)
Sensory deficits extremities	2 (40.0)	0 (0.0)	0 (0.0)	0 (0.0)	0 (0.0)	7 (58.3)	3 (25.0)	1 (8.3)	1 (8.3)	1 (8.3)	13 (65.0)	7 (35.0)	5 (26.3)	5 (25.0)	2 (15.4)
Motor deficits extremities	1 (20.0)	0 (0.0)	0 (0.0)	0 (0.0)	1 (20.0)	3 (25.0)	3 (25.0)	3 (25.0)	1 (8.3)	1 (8.3)	3 (15.0)	6 (30.0)	6 (31.6)	5 (25.0)	4 (30.8)
Reflex asymmetries	0 (0.0)	0 (0.0)	0 (0.0)	0 (0.0)	0 (0.0)	0 (0.0)	0 (0.0)	0 (0.0)	0 (0.0)	0 (0.0)	0 (0.0)	1 (5.0)	2 (10.5)	1 (5.0)	0 (0.0)
Pyramidal signs	0 (0.0)	0 (0.0)	0 (0.0)	1 (25.0)	1 (20.0)	0 (0.0)	0 (0.0)	1 (8.3)	0 (0.0)	0 (0.0)	1 (5.0)	3 (15.0)	4 (21.1)	2 (10.0)	2 (15.4)

Data are patient numbers (%).

Table S3. T scores of individual neuropsychological tests in thalamic stroke patients and their matched controls

**Neuropsychological assessment**	Total cohort of control patients (n=37)	Total cohort of thalamic stroke patients (n=37)	Matched controls anterior thalamic stroke (n=5)	Anterior thalamic stroke patients (n=5)	Matched controls paramedian thalamic stroke (n=12)	Paramedian thalamic stroke patients (n=12)	Matched controls inferolateral thalamic stroke (n=20)	Inferolateral thalamic stroke patients (n=20)
**Digit span forwards**								
1 month	51.8 (42.9;61.8)	49.5 (41.2;51.8) ***0.041**	57.1 (40.2;66.2)	41.6 (39.3;49.5)	44.3 (39.0;56.7)	51.2 (47.1;59.2)	57.1 (49.8;66.5)	42.9 (39.6;50.8) ***0.020, †0.002, #0.024**
6 months	55.0 (45.9;65.6)	46.1 (40.6;53.5) ***0.003**	50.8 (35.5;60.6)	46.4 (35.2;49.5) ***0.024**	52.6 (44.6;69.1)	50.1 (40.7;66.5)	57.1 (54.4;65.3)	44.2 (39.6;50.8) ***0.002, †<0.001**
12 months	57.1 (45.0;66.5)	49.5 (41.2;55.0) ***0.005**	57.1 (29.5;68.5)	46.2 (39.7;55.6)	50.1 (44.6;59.6)	47.7 (41.8;55.0)	64.1 (49.8;70.5)	50.8 (40.1; 54.8) ***0.024, †0.006**
24 months	52.6 (44.8;64.3)	44.5 (38.7;56.0) ***0.015**	54.4 (42.9;68.5)	41.2 (38.5;41.6) ***0.011, †0.015**	50.8 (41.6;64.8)	56.0 (49.8;57.3)	55.7 (49.8;62.6)	39.6 (33.6;50.1) ***0.002, †0.002, #0.006**
**Digit span backwards**								
1 month	54.4 (44.2;61.3)	45.9 (38.3;50.8) ***0.001**	45.9 (36.7;59.0)	38.3 (35.2;42.6) ***0.004**	48.3 (44.8;56.4)	42.5 (38.3;54.3)	57.7 (44.6;64.6)	46.4 (38.4;50.8) ***0.018, †0.010**
6 months	50.8 (45.2;64.8)	45.9 (39.2;50.8) ***0.008**	50.8 (38.5;60.1)	44.8 (36.7;50.7)	46.9 (44.8;60.6)	46.4 (40.4;50.7)	52.6 (46.9;64.8)	45.9 (38.7;50.8) ***0.024, †0.008**
12 months	50.8 (45.9;62.7)	46.9 (38.7;50.8) ***0.011**	50.5 (48.7;55.6)	46.9 (36.7;46.9) **†0.034**	54.1 (44.8;60.6)	48.1 (44.1;53.5)	54.2 (44.4;64.6)	46.4 (38.7;53.5) ***0.031**
24 months	54.4 (46.9;61.0)	46.9 (41.3;54.4) ***0.005**	47.5 (38.3;62.2)	38.3 (36.5;43.1) ***0.006, †0.011**	54.4 (45.9;61.3)	50.0 (44.8;55.1)	55.0 (50.8;62.2)	50.8 (41.3;54.4) ***0.044, †0.020**
**RBMT immediate recall**								
1 month	49.3 (42.4;56.9)	44.3 (40.5;50.0) ***0.029**	55.7 (41.1;62.6)	40.5 (31.0;46.8) ***0.021, †0.094**	53.1 (44.6;58.5)	50.6 (43.7;55.7)	46.2 (42.1;54.4)	44.3 (40.5;49.0) ***0.043**
6 months	50.6 (46.2;55.7)	40.5 (31.0;46.8)	55.7 (43.0;56.3)	40.5 (36.1;50.6) **†0.112**	53.8 (49.3;59.8)	44.9 (32.9;54.1)	48.7 (45.9;51.6)	48.1 (43.0;55.7)
12 months	50.6 (44.3;55.0)	44.3 (40.5;49.0)	56.9 (51.9;63.2)	44.3 (38.0;48.1) **†0.009**	50.6 (44.3;55.0)	53.1 (44.3;61.7)	48.1 (43.3;53.1)	46.2 (42.1;57.6)
24 months	53.1 (44.6;57.9)	46.8 (39.9;58.8)	54.4 (39.9;60.1)	44.9 (32.9;54.1)	55.0 (44.3;55.7)	51.2 (47.1;55.7)	46.8 (41.8;56.3)	50.6 (43.7;55.0)
**RBMT delayed recall**								
1 month	49.8 (44.8;60.2)	43.6 (39.9;51.6) ***0.002**	51.0 (44.2;67.0)	39.9 (30.7;43.6) ***0.002, †0.015**	54.1 (46.7;59.3)	48.5 (41.4;57.1) **‡0.034**	46.7 (44.8;59.0)	42.4 (38.7;49.4) ***0.004**
6 months	52.2 (47.9;57.8)	47.9 (42.7;53.1) ***0.015**	47.3 (43.6;60.2)	39.9 (36.8;49.1) ***0.009**	55.9 (50.4;60.2)	51.0 (43.6;58.7)	49.8 (47.6;56.8)	47.3 (43.6;52.2) ***0.025**
12 months	49.8 (46.1;57.1)	46.1 (42.4;55.9)	58.4 (52.2;65.1)	37.4 (35.0;43.6) ***0.002, †0.009**	49.1 (46.7;52.8)	50.4 (42.4;59.3) **‡0.019**	48.5 (44.8;54.7)	46.1 (42.7;55.9) **‡0.020**
24 months	51.0 (45.1;58.1)	49.8 (45.4;55.3)	55.9 (39.3;57.2)	45.4 (36.5;54.4)	58.4 (54.7;60.8)	51.0 (48.5;58.1)	47.3 (43.9;50.7)	49.8 (45.4;54.1)

**Block span forwards**								
1 month	51.8 (40.1;58.9)	51.8 (44.2;55.3)	52.0 (47.7;57.4)	35.2 (35.2;57.9)	56.3 (38.1;59.6)	50.0 (43.5;63.7)	50.9 (36.0;62.2)	52.0 (44.2;55.5)
6 months	52.0 (39.6;60.4)	50.0 (43.9;57.1)	51.8 (46.6;54.7)	52.0 (44.6;60.4)	57.2 (45.6;69.5)	50.9 (45.6;59.6)	48.0 (36.0;60.4)	44.2 (43.3;58.1)
12 months	55.5 (44.2;62.8)	51.8 (43.3;57.4)	55.5 (51.0;67.3)	51.8 (35.2;60.4)	56.5 (45.4;65.5)	55.5 (44.5;59.6)	51.0 (43.9;59.6)	51.8 (43.3;57.4)
24 months	50.0 (43.9;59.6)	51.8 (43.3;56.5)	57.4 (51.0;66.5)	48.7 (44.2;65.9)	44.2 (35.2;57.1)	50.0 (37.8;56.9)	47.1 (43.9;60.4)	51.8 (44.2;57.9)
**Block span backwards**								
1 month	45.0 (43.9;54.8)	44.5 (38.7;55.2)	47.5 (43.8;59.0)	42.3 (42.3;53.3)	46.3 (37.7;58.3)	44.5 (38.0;63.7)	43.9 (37.7;53.0)	47.5 (37.7;55.2)
6 months	47.5 (43.9;54.8)	48.7 (42.3;55.2)	51.3 (42.6;57.4)	54.7 (43.0;57.8)	52.8 (47.8;62.6)	53.2 (39.3;56.4)	43.9 (42.7;48.4)	47.5 (41.1;52.3)
12 months	47.5 (42.3;59.2)	48.7 (42.3;55.2)	43.9 (40.0;63.4)	48.7 (42.4;57.8)	50.0 (42.7;57.1)	49.4 (39.6;56.6)	45.7 (42.7;59.8)	47.5 (44.2;55.0)
24 months	47.5 (43.9;54.4)	48.7 (43.9;56.7)	43.9 (40.0;63.4)	55.0 (41.6;59.2)	47.5 (42.3;52.0)	52.8 (42.7;57.7)	48.1 (43.9;55.2)	43.9 (43.9;54.8)
**RWT categories**								
1 month	62.8 (52.8;70.5)	50.3 (41.6;56.1)<br>***<0.001**	54.7 (51.4;68.7)	36.6 (30.3;48.4)<br>***0.003, †0.047**	63.4 (50.9;72.6)	50.9 (42.8;66.4)<br>***0.035**	61.4 (52.7;70.1)	52.8 (43.0;57.0)<br>***0.002, †0.008, ‡0.030**
6 months	59.5 (49.5;70.5)	50.0 (44.8;59.7)<br>***0.023**	56.7 (50.0;65.8)	41.2 (40.0;51.3)<br>***0.016**	63.1 (54.6;72.6)	50.0 (46.8;60.4)<br>**‡0.035**	61.2 (48.2;68.9)	54.4 (46.9;62.3)<br>**‡0.039**
12 months	60.4 (51.2;68.8)	51.8 (43.0;57.7)<br>***0.001**	53.9 (45.3;65.2).	42.6 (35.8;51.8)<br>***0.005**	64.4 (52.8;72.6)	57.1 (45.9;62.3)<br>**‡0.027**	60.0 (52.0;67.8)	51.6 (43.1;65.8)<br>***0.003, †0.007**
24 months	60.4 (53.8;70.5)	55.0 (44.8;65.8)<br>***0.029**	58.4 (41.6;68.0)	38.8 (30.3;49.8)<br>***0.005**	65.6 (55.2;73.3)	56.5 (47.1;70.1)<br>**‡0.025**	60.4 (53.8;70.5)	55.2 (50.0;65.8)<br>**‡0.017**
**RWT letters**								
1 month	54.4 (44.7;58.4)	41.0 (36.0;51.5)<br>***<0.001**	58.1 (47.9;60.1)	32.5 (29.0;39.3)<br>***0.001, †0.016**	53.3 (44.1;57.8)	42.8 (36.7;50.5)<br>***0.002, †0.033, ‡0.020**	54.0 (44.5;59.6)	45.3 (37.4;52.6)<br>***0.002, †0.005, ‡0.014**
6 months	52.3 (42.5;61.5)	44.0 (37.2;50.4)<br>***0.003**	57.7 (33.8;68.7)	38.7 (26.7;44.0)<br>***0.018, †0.169**	53.2 (44.4;60.0)	47.5 (37.6;57.9)<br>**‡0.045**	51.4 (39.7;61.6)	44.2 (37.2;49.5)<br>***0.009, †0.035**
12 months	52.8 (44.5;57.2)	43.4 (38.1;54.7)<br>***0.019**	55.0 (43.7;71.0)	33.6 (27.9;40.4)<br>***0.005, †0.014**	56.0 (45.8;56.4)	41.5 (38.1;60.6)	50.8 (43.2;59.0)	47.1 (41.1;54.7)<br>**‡0.022**
24 months	55.5 (45.5;62.3)	45.3 (41.9;53.3)<br>***0.006**	54.7 (37.2;71.9)	39.8 (35.9;42.4)<br>***0.005, †0.042**	55.8 (42.9;60.4)	46.9 (43.9;52.5)<br>**‡0.015**	56.3 (48.0;62.8)	45.3 (41.9;54.1)<br>***0.034, †0.021**
**TMT part A**								
1 month	56.7 (46.9;60.4)	46.8 (39.6;56.7)<br>***0.008**	56.7 (47.0;58.6)	50.0 (38.4;55.8)	60.4 (52.1;62.8)	46.8 (40.5;56.7)	56.0 (43.7;59.5)	46.2 (39.6;58.4)
6 months	55.2 (48.1;62.8)	48.7 (41.6;56.7)<br>***0.038**	52.5 (40.2;61.6)	51.2 (45.4;55.5)	54.5 (50.0;62.8)	54.6 (44.8;62.2)	55.2 (46.5;62.8)	46.2 (39.6;54.6)
12 months	53.9 (46.2;60.4)	56.0 (45.4;60.4)	58.4 (41.0;61.6)	58.4 (43.0;61.6)	55.3 (48.1;62.8)	56.7 (49.4;60.4)	53.9 (44.8;59.6)	51.3 (37.2;56.7)
24 months	55.2 (47.8;60.4)	53.9 (42.4;60.4)	48.7 (44.5;59.8)	49.4 (40.1;59.6)	55.2 (50.0;62.8)	56.7 (45.3;60.4)	58.4 (46.8;60.4)	51.3 (40.6;59.4)

**TMT part B**								
1 month	55.2 (46.2;60.4)	46.2 (39.4;54.5) ***0.011**	55.2 (41.7;59.8)	37.2 (37.2;41.7) ***0.003, †0.044**	53.2 (45.1;59.5)	46.2 (39.6;49.7) ***0.047; ‡0.031**	56.7 (46.8;60.4)	50.0 (40.5;59.5) **‡0.009**
6 months	52.5 (43.3;62.8)	48.7 (39,6;54.4)	53.9(38.4;62.8)	37.2 (37.2;40.2) ***0.005**	58.6 (50.4;62.8)	56.0 (47.8;59.5) **‡0.005**	50.0 (40.8;60.6)	48.7 (39.6;57.2) **‡0.015**
12 months	52.5 (48.1;60.4)	50.6 (43.3;60.4)	51.3 (48.7;58.3)	39.6 (37.2;41.4) ***0.002; †0.008**	56.7 (51.3;60.4)	55.3 (48.7;59.5) **‡0.006**	52.5 (41.3;60.4)	51.3 (47.5;60.4) **‡0.008**
24 months	53.9 (48.7;60.4)	56.7 (41.4;60.4)	53.9 (52.6;58.6)	37.2 (37.2;54.6)	60.4 (48.7;62.8)	58.6 (48.8;60.4)	52.6 (48.7;60.4)	53.9 (41.4;60.4)
**FWIT Stroop test**								
1 month	55.0 (48.8;65.0)	50.5 (43.3;61.0) ***<0.001**	55.0 (50.8;68.5)	44.0 (37.8;51.0) ***0.001, †0.016**	57.0 (47.5;66.3)	51.3 (44.8;57.5) ***0.012, †0.028, ‡0.006**	57.0 (46.6;65.0)	55.0 (43.0;63.0) **‡0.001**
6 months	57.0 (51.0;66.0)	52.0 (45.5;61.0) ***0.003**	52.5 (49.3;65.0)	43.5 (40.5;49.3) ***<0.001, †0.016**	55.5 (50.0;69.8)	53.0 (46.9;60.5) **‡0.009**	60.5 (51.4;65.5))	52.8 (46.0;52.3) ***0.044, ‡0.012**
12 months	59.0 (49.0;66.5)	52.0 (43.6;62.0) ***0.002**	53.0 (52.3;66.0)	45.5 (42.8;49.5) ***0.007, †0.008**	56.5 (47.9;66.8)	56.0 (46.8;61.8)	61.0 (48.6;66.8)	54.0 (43.0;64.0) **† 0.021, *0.015**
24 months	54.5 (49.0;64.8)	52.0 (45.0;63.0) ***0.020**	52.0 (49.8;54.5)	44.8 (41.0;47.0) ***0.002, †0.050**	54.0 (46.5;64.0)	55.0 (47.9;63.5) **‡0.042**	58.5 (51.3;68.0)	53.0 (45.0;64.5) **† 0.045, ‡0.023**
**TAP alertness without warning tone**								
1 month	44.0 (39.0;52.0)	41.5 (36.0;47.8)	44.0 (33.5;55.0)	41.0 (37.5;52.0)	47.0 (39.0;52.8)	40.0 (37.0;48.0)	43.0 (39.3;51.5)	44.0 (35.0;47.0)
6 months	43.0 (39.0;50.0)	45.0 (38.0;49.0	50.0 (42.5;55.5)	45.0 (38.0;60.0)	45.0 (39.3;49.5)	48.0 (38.8;52.8)	42.0 (37.3;48.3)	41.0 (37.3;48.0)
12 months	45.0 (38.3;54.8)	42.0 (38.3;48.0)	46.0 (33.8;53.8)	47.0 (33.0;53.5)	45.5 (38.3;53.0)	45.0 (39.0;52.8)	43.0 (37.5;55.0)	39.0 (33.0;46.0)
24 months	43.0 (37.3;52.8)	43.0 (35.5;51.5)	38.0 (30.5;51.0)	46.5 (39.0;52.5)	43.0 (39.0;50.0)	44.0 (35.3;54.5)	43.5 (36.5;56.0)	43.0 (33.0;47.0)
**TAP alertness with warning tone**								
1 month	45.0 (37.0;50.0)	41.0 (35.3;47.8)	39.0 (38.0;48.5)	41.0 (38.0;54.0)	47.0 (36.3;55.3)	41.5 (36.0;47.0)	43.5 (37.3;48.5)	41.0 (33.0;47.0)
6 months	44.0 (38.5;48.0)	43.0 (37.0;48.0)	46.0 (43.0;49.0)	45.0 (38.5;55.0)	42.5 (38.3;50.3)	44.0 (42.3;47.8)	42.5 (37.3;48.0)	39.5 (35.0;46.5)
12 months	42.5 (37.0;49.0)	43.0 (36.3;46.8)	41.5 (38.8;48.0)	44.0 (39.0;49.0)	40.5 (34.8;49.0)	45.0 (42.0;50.8)	45.0 (36.3;52.0)	40.0 (33.0;44.0)
24 months	42.5 (37.5;48.0)	43.0 (35.0;48.0)	39.0 (36.0;44.5)	46.0 (37.0;53.5)	41.0 (36.0;46.0)	43.5 (39.0;49.8)	45.0 (39.0;53.5)	41.0 (34.0;43.5)
**TAP Go/No-go reaction time**								
1 month	49.0 (45.0;57.0)	49.0 (40.0;56.0)	49.0 (44.5;61.0)	49.0 (44.0;54.0)	52.5 (48.0;57.8)	49.5 (38.3;58.3)	49.0 (39.0;56.0)	49.5 (39.8;57.5)
6 months	49.0 (44.0;58.5)	49.0 (40.0;59.0)	44.0 (39.0;53.0)	51.0 (45.5;62.5)	51.0 (44.8;59.0)	45.0 (39.3;55.5)	49.0 (45.0;59.3)	49.0 (39.0;60.3)
12 months	50.0 (43.5;56.0)	48.0 (42.5;56.8)	50.0 (43.0;58.5)	57.0 (47.5;63.5)	50.5 (46.3;57.5)	47.5 (42.5;55.5)	50.0 (39.8;55.8)	50.0 (39.0;54.0)
24 months	50.0 (43.5;57.5)	50.0 (40.0;58.0)	45.0 (39.0;48.5)	52.0 (45.0;60.5)	51.0 (49.0;55.0)	46.5 (40.0;50.0) **†0.031**	52.5 (42.3;60.5)	54.0 (38.5;61.0)

**TAP divided attention auditive**								
1 month	46.0 (35.3;52.0)	40.5 (31.3;49.0)	51.0 (33.5;60.0)	45.0 (32.0;50.5)	48.0 (43.3;52.0)	43.0 (32.0;49.0)	43.0 (34.0;48.0)	36.0 (27.8;46.5)
6 months	45.0 (37.0;52.0)	41.0 (29.0;47.3)	56.0 (36.0;61.0)	46.0 (28.5;54.5)	49.0 (41.5;54.3)	42.5 (31.0;47.8)	38.5 (34.0;48.3)	38.0 (29.0;45.5)
12 months	43.0 (38.0;51.0)	41.0 (35.0;49.0)	43.0 (38.0;57.0)	45.0 (32.5;52.0)	48.0 (43.8;51.5)	44.5 (37.3;48.5)	39.5 (36.5;49.5)	38.5 (32.0;48.8)
24 months	49.0 (36.0;53.0)	40.0 (30.5;49.0)	41.0 (34.5;52.5)	53.5 (38.3;59.8)	51.0 (38.0;59.0)	45.5 (29.0;52.8)	42.0 (35.0;53.0)	37.0 (30.5;40.5)
**TAP divided attention visual**								
1 month	55.5 (48.5;60.0)	54.0 (48.0;58.8)	56.0 (42.5;63.5)	55.0 (52.0;59.5)	52.5 (48.5;59.0)	56.5 (50.5;58.5)	57.0 (51.0;60.0)	51.0 (44.0;59.0)
6 months	58.0 (50.0;63.0)	50.0 (42.0;58.0) *0.012	57.0 (41.5;71.0)	52.0 (49.5;58.5)	56.0 (50.8;62.3)	50.0 (40.3;64.3)	59.0 (52.0;66.5)	50.5 (40.8;55.8) †0.014
12 months	58.0 (51.3;63.0)	53.5 (48.3;58.0) *0.050	62.0 (49.0;63.5)	55.0 (49.0;57.5)	57.0 (51.5;62.0)	54.0 (49.3;58.8)	59.0 (46.0;65.0)	52.0 (45.0;59.0)
24 months	56.0 (53.0;59.0)	55.0 (50.0;60.0)	54.0 (41.0;59.5)	56.0 (43.8;61.5)	55.0 (54.0;56.0)	57.5 (50.0;64.3)	57.0 (53.0;65.0)	54.0 (50.5;56.5)
**TAP divided attention total**								
1 month	46.0 (41.0;54.5)	51.0 (41.0;55.0)	53.0 (43.5;60.0)	51.0 (34.0;55.0)	47.5 (41.5;54.3)	52.5 (40.3;59.0)	46.0 (41.0;52.0)	51.0 (41.0;52.3)
6 months	48.0 (41.0;52.0)	48.5 (43.5;53.0)	52.0 (48.0;56.5)	50.0 (39.5;56.0)	47.0 (41.0;54.3)	44.0 (41.8;49.3)	46.5 (38.0;51.3)	52.0 (44.5;59.0)
12 months	46.5 (43.0;52.9)	50.0 (45.0;59.0)	52.0 (46.5;60.5)	59.0 (42.0;61.0)	51.5 (45.3;58.0)	50.0 (44.0;54.5)	44.0 (41.0;51.0)	49.0 (45.0;59.5)
24 months	51.0 (44.0;53.0)	48.0 (40.0;59.0)	60.0 (55.5;63.0)	60.0 (49.3;61.8)	45.0 (43.0;51.0)	48.0 (38.0;58.0)	51.0 (44.0;52.0)	45.0 (38.5;56.5)

Data are medians (Q1;Q3). *p-value vs total cohort of controls, †p-value vs corresponding matched controls, ‡p-value vs anterior thalamic stroke patients, #p-value vs paramedian thalamic stroke patients.

Table S4. Raw data of individual neuropsychological tests in thalamic stroke patients and their matched controls

**Neuropsychological assessment**	Total cohort of control patients (n=37)	Total cohort of thalamic stroke patients (n=37)	Matched controls anterior thalamic stroke (n=5)	Anterior thalamic stroke patients (n=5)	Matched controls paramedian thalamic stroke (n=12)	Paramedian thalamic stroke patients (n=12)	Matched controls inferolateral thalamic stroke (n=20)	Inferolateral thalamic stroke patients (n=20)
**Digit span forwards (number)**								
1 month	8.0 (6.0;10.0)	7.0 (6.0;8.0)<br>***0.045**	8.0 (6.0;11.5)	6.0 (5.5;7.5)	6.5 (6.0;8.5)	8.0 (7.0;9.5)	8.0 (7.0;10.8)	6.0 (5.3;7.8)<br>***0.011, †0.002, #0.009**
6 months	8.0 (6.5;10.0)	7.0 (6.0;8.0)<br>***0.004**	6.0 (5.0;9.5)	7.0 (5.0;7.0)<br>***0.029**	8.0 (6.3;11.0)	8.0 (6.0; 10.8)	8.0 (8.0;10.0)	6.0 (6.0;7.0)<br>***0.001, †<0.001**
12 months	8.0 (6.5;11.0)	7.0 (6.0;8.0)<br>***0.007**	8.0 (4.0;11.0)	7.0 (5.5;8.5)	8.0 (6.3;9.0)	7.0 (6.3; 8.8)	9.5 (7.0;11.0)	7.0 (5.3;8.0)<br>***0.015, †0.006**
24 months	8.0 (7.0;11.0)	7.0 (5.5;8.0)<br>***0.017**	9.0 (7.0;11.0)	6.0 (5.3;6.0)<br>***0.010, †0.029**	7.0 (6.0;11.0)	8.0 (7.0;9.0)<br>**‡0.013**	8.0 (7.0;10.5)	6.0 (4.5;7.0)<br>***0.001, †0.002, #0.002**
**Digit span backwards (number)**								
1 month	6.0 (5.5;9.0)	6.0 (4.5;6.5)<br>***0.003**	6.0 (4.0;7.0)	5.0 (3.5;5.5)<br>***0.012**	6.0 (6.0;8.0)	5.5 (5.0;7.8)	7.5 (5.3;10.0)	6.0 (4.3;6.8)<br>***0.018, †0.010**
6 months	6.0 (6.0;9.5)	6.0 (5.0;6.8)<br>***0.010**	6.0 (4.5;9.0)	6.0 (3.5;6.5)	6.0 (6.0;9.3)	6.0 (5.0;7.0)	7.0 (6.0;9.8)	6.0 (5.0;6.0)<br>***0.015, †0.008**
12 months	7.0 (6.0;9.0)	6.0 (4.5;7.0)<br>***0.006**	7.0 (6.0;7.5)	6.0 (4.5;6.0)	7.0 (6.0;8.8)	6.0 (5.25;7.0)	6.5 (5.3;10.0)	6.0 (4.0;6.75)<br>***0.011**
24 months	7.0 (6.0;9.0)	6.0 (5.0;7.0)<br>***0.009**	7.0 (5.0;9.0)	5.0 (4.5;6.0)<br>***0.012**	7.0 (6.0;9.0)	6.5 (6.0;7.0)<br>**‡0.013**	7.5 (6.0;8.8)	6.0 (5.0;7.0)<br>***0.022, †0.020**
**RBMT immediate recall (number)**								
1 month	9.5 (6.8;12.5)	7.5 (6.0;9.8)<br>***0.026**	12.0 (6.5;14.8)	6.0 (2.3;8.5)<br>***0.018**	11.0 (7.6;13.1)	8.5 (5.8;13.3)	8.3 (6.6;11.5)	7.5 (6.0;9.4)<br>***0.039**
6 months	10.0 (8.8;12.0)	9.0 (6.1;12.0)	12.0 (9.0;12.3)	6.0 (4.3;10.0)<br>***0.050, †0.046**	11.3 (9.5;13.6)	10.0 (6.25;13.4)	9.3 (8.1;10.4)	9.0 (7.0;12.0)
12 months	10.5 (8.3;11.8)	9.0 (7.0;12.8)	12.5 (7.5;15.0)	7.5 (5.0;9.0)	10.0 (7.5;11.8)	11.0 (7.5;14.4)	9.0 (7.1;11.0)	8.3 (6.6;12.8)
24 months	11.0 (7.6;12.9)	10.0 (7.3;12.0)	11.5 (5.5;13.8)	7.8 (3.0;11.4)	12.0 (11.0;13.5)	10.3 (8.6;12.0)	8.5 (6.5;12.3)	10.0 (7.3;11.8)
**RBMT delayed recall (number)**								
1 month	8.5 (6.5;12.8)	6.0 (4.5;9.25)<br>***0.002**	9.0 (6.0;15.5)	4.5 (0.8;6.0)<br>, ***0.002, †0.028**	10.3 (7.3;12.4)	8.0 (5.1;11.5)<br>**‡0.034**	7.3 (6.5;12.3)	5.5 (4.0;8.4)<br>***0.004, †0.034**
6 months	9.5 (8.0;11.8)	7.8 (5.6;9.9)<br>***0.009**	9.0 (7.3;12.8)	4.5 (3.3;8.3)<br>***0.005**	11.0 (8.8;12.8)	9.0 (6.0;12.1)	8.5 (7.6;11.4)	7.5 (6.0;9.5)<br>***0.014**
12 months	8.0 (6.8;10.8)	7.0 (5.5;11.0)	12.0 (5.8;14.8)	3.5 (2.5;6.0)<br>***0.001, †0.028**	8.3 (7.3;9.8)	8.8 (5.5;12.4)<br>**‡0.019**	8.0 (6.5;10.5)	7.0 (5.6;11.0)<br>**‡0.019**
24 months	9.3 (6.9;11.6)	8.5 (6.8;10.8)	11.0 (5.3;11.5)	6.8 (3.1; 10.4)	12.0 (10.5;13.0)	9.0 (8.0;11.9)	7.5 (6.1;8.9)	8.5 (6.8;10.3)

**Block span forwards (number)**								
1 month	8.0 (7.0;9.5)	8.0 (7.0;9.0)	8.0 (7.5;8.5)	6.0 (5.5;10.0)	9.0 (5.8;10.0)	8.0 (7.0;10.5)	8.0 (6.3;9.8)	8.0 (7.0; 9.0)
6 months	9.0 (6.5;9.0)	8.0 (7.0;9.0)	8.0 (7.5;8.5)	8.0 (7.5;9.0)	9.0 (7.3;11.0)	8.0 (7.3;9.0)	7.5 (6,0;9.0)	7.0 (7.0;9.0)
12 months	8.0 (7.0;10.0)	8.0 (7.0;9.0)	8.0 (7.0;10.0)	9.0 (6.0;9.0)	9.0 (6.5;10.0)	9.0 (7.3;9.8)	8.0 (7.0;9.0)	8.0 (7.0; 9.0)
24 months	8.0 (7.0;9.0)	8.0 (7.0;9.0)	8.0 (7.5;9,5)	8.0 (7.3;11.0)	7.0 (6.0;10.0)	8.5 (6.3; 9.0)	7.5 (7.0;9.0)	8.0 (7.0; 9.0)
**Block span backwards (number)**								
1 month	7.0 (6.0;8.5)	7.0 (6.0;8.0)	7.0 (6.5;7.5)	6.0 (6.0;8.5)	7.0 (6.0;9.0)	6.5 (6.0;9.8)	6.0 (6.0;7.8)	7.0 (6.0;8.0)
6 months	7.0 (6.0;8.5)	7.0 (6.0;9.0)	8.0 (7.0;9.5)	8.0 (6.0;9.0)	8.0 (7.0;10.0)	8.0 (6.0;9.0)	6.0 (6.0;7.0)	7.0 (6.0;7.25)
12 months	7.0 (6.0;9.0)	7.0 (6.0;8.8)	6.0 (6.0;9.0)	7.0 (6.5;8.5)	7.5 (6.0;9.0)	8.0 (6.0;9.0)	6.5 (6.0;9.0)	7.0 (7.0;8.0)
24 months	7.0 (6.0;8.0)	7.0 (6.0;9.0)	6.0 (6.0;9.0)	8.5 (5.8;9.0)	7.0 (6.0;9.0)	8.0 (6.0;10.0)	7.0 (6.0;8.0)	6.0 (6.0;8.0)
**TMT part A (seconds)**								
1 month	28.0 (22.0;36.5)	36.0 (24.3;44.8)	26.0 (23.0;40.5)	32.0 (23.0;43.5)	22.5 (19.3;33.8)	35.0 (23.8;40.5)	32.0 (24.0;43.3)	39.0 (24.0;60.0) ***0.046**
6 months	29.0 (21.5;35.5)	34.0 (23.0;43.0)	35.0 (24.5;45.0)	31.0 (23.5;34.0)	24.5 (19.3;33.8)	28.0 (17.3;36.3)	29.5 (24.0;41.0)	41.0 (28.75;57.25) ***0.017, #0.025**
12 months	29.0 (21.5; 40.0)	30.0 (24.0;43.0)	34.0 (22.0;42.0)	24.0 (21.0;37.0)	25.0 (19.0;32.3)	25.5 (19.8;31.5)	32.5 (24.5;45.3)	38.0 (26.0;51.0) **#0.043**
24 months	28.0 (22.3; 34.8)	32.0 (23.0;42.0)	34.0 (21.5;40.5)	29.5 (23.25;40.25)	27.0 (19.0;33.0)	25.5 (19.3; 38.0)	28.0 (23.3;35.3)	37.0 (26.5;57.5)
**TMT part B (seconds)**								
1 month	64.0 (53.5;88.0)	80.0 (61.0;114.0)	82.0 (55.5;95.5)	108.0 (83.0;156.0) ***0.018**	61.0 (54.0;67.8)	68.0 (61.5; 97.8)	68.5 (53.3;89.8)	83.0 (51.0;133.0)
6 months	68.0 (49.5;83.5)	80.0 (58.0;126.0)	68.0 (51.0; 76.5)	126 (93.5;140.5) ***0.023, †0.008**	56.5 (42.5;67.5)	58.5 (42.5;89.8) **‡0.014**	78.5 (62.3;138.5)	101.5 (63.8;131.8) ***0.044, #0.015**
12 months	68.0 (48.0;82.0)	68.5 (57.3;98.3)	66.0 (45.5;68.0)	89.0 (80.5;127.0) ***0.018, †0.008**	50.5 (44.3;77.8)	59.0 (46.8;66.0) **‡0.006**	71.0 (60.5;102.8)	72.0 (65.0;110.0) **#0.014**
24 months	64.0 (49.8;87.3)	65.0 (53.0;100.5)	71.0 (57.0;85.0)	104 (62.75;124.25)	53.0 (43.0;70.0)	54.0 (45.8;68.8)	72.5 (52.5;102.8)	89.0 (64.0;116.0) ***0.040, #0.014**
**Stroop test (seconds)**								
1 month	77.0 (61.0;90.0)	88.5 (78.3;117.3) ***0.002**	79.0 (62.0;103.5)	135.0 (106.0;214.5) ***<0.001, †0.028**	77.0 (61.3;85.5)	82.5 (78.25;94.5) **‡0.006**	74.5 (61.0;97.8)	85.0 (75.0;120.0) ***0.027, ‡0.024**
6 months	75.0 (58.5;90.5)	85.0 (69.0;106.0) ***0.026**	87.0 (61.5;93.0)	105.0 (99.5;126.5) ***0.002, †0.008**	68.0 (59.5;82.8)	77.0 (65.25;85.5) **‡0.006**	75.5 (58.3;102.0)	87.0 (68.8;114.0) ***0.040**

12 months	71.0 (59.5; 87.5)	83.0 (71.3;100.8) ***0.016**	77.0 (60.0;87.5)	92.0 (89.0;99.5) ***0.029, †0.028**	70.5 (66.5;81.3)	74.5 (62.75;88.25)	70.5 (56.3;102.5)	84.0 (71.0;110.0) ***0.013, †0.044**
24 months	74.5 (61.3;88.3)	77.0 (70.5;94.5)	86.0 (65.5;93.5)	95.5 (90.75;114.5) ***0.007**	74.0 (62.0;84.0)	73.0 (61.75;88.5) **‡0.042**	69.0 (59.5;87.8)	77.0 (70.0;94.5) **†0.036**
**TAP alertness without warning tone (seconds)**								
1 month	254.0 (231.0;305.0)	267.5 (243.8;333.5)	244.0 (210.0;326.5)	263.0 (228.0;290.0)	250.5 (233.0;310.0)	266.0 (240.75;338.8)	260.5 (230.5;305.0)	269.0 (243.0;359.0)
6 months	256.0 (234.0;292.0)	256.0 (234.0;312.0)	235.0 (213.0;263.0)	243.0 (202.5;291.5)	256.0 (234.0;286.5)	244.5 (226.5;281.8)	274.5 (243.0;337.8)	291.5 (239.75;324.5)
12 months	253.0 (223.0;312.0)	267.5 (236.5;322.3)	243.0 (218.5;305.0)	240.0 (222.0;371.0)	253.5 (224.5;299.8)	247.0 (228.0;288.0)	261.5 (224.5;327.0)	296.0 (240.0;332.0) ***0.039**
24 months	260.0 (222.8;302.5)	264.0 (233.5;323.0)	253.0 (219.5;340,5)	248.5 (219.5;288.0)	262.0 (253.0;301.0)	268.5 (227.0;321.8)	254.5 (220.5;315.5)	274.0 (250.0;382.5)
**TAP alertness with warning tone (seconds)**								
1 month	250.0 (225.0;293.0)	264.0 (234.3;325.3)	270.0 (212.5;287.5)	260.0 (209.0;278.0)	235.5 (218.8;292.5)	255.5 (233.3;325.8)	255.0 (239.5;294.5)	271.0 (243.0;355.0)
6 months	249.0 (223.5;281.5)	252.0 (231.0;299.0)	226.0 (205.0;253.5)	239.0 (207.0;278.5)	248.5 (235.3;285.0)	251.0 (229.75;273.5)	257.0 (220.3;298.3)	293.0 (231.8;323.3)
12 months	253.0 (220.0;308.5)	252.0 (233.5;305.8)	257.0 (211.5;271.5)	249.0 (223.5;274.0)	255.5 (226.0;317.5)	241.5 (215.75;262.3)	250.0 (219.3;309.8)	284.0 (248.0; 339.0) ***0.032, †0.050, #0.025**
24 months	252.5 (222.8;285.5)	247.0 (232.0;318.5)	265.0 (219.0;289.0)	237.0 (213.25;296.0)	254.0 (232.0;307.0)	245.5 (221.5;280.0)	245.0 (215.5;283.0)	274.0 (245.0;348.5)
**TAP Go/No-go reaction time (seconds)**								
1 month	424.5 (383.5;472.3)	420.0 (387.0;499.0)	406.0 (378.0;470.5)	407.0 (393.0;464.5)	402.0 (376.0;440.3)	415.5 (359.5;544.3)	433.0 (393.0;500.0)	424.0 (390.0;512.3)
6 months	430.0 (389.5;457.5)	430.0 (359.0;496.0)	469.0 (423.0;514.5)	413.0 (340.5;456.5)	409.0 (386.3;445.8)	445.0 (360.0;501.0)	442.0 (382.3;457.8)	432.0 (363.5;535.5)
12 months	422.0 (398.0;463.0)	439.0 (368.5;467.0)	440.0 (401.0;490.5)	361.0 (346.5;443.0)	409.5 (395.3;439.5)	432.0 (363.0;466.3)	422.5 (408.3;508.5)	452.0 (392.0;494.0)
24 months	409.0 (383.85;467.5)	420.0 (381.5;503.5)	463.0 (394.0;520.5)	412.0(350.75;458.25)	399.0 (386.0;430.0)	444.0 (408.0; 510.3)	416.5 (364.0;469.0)	411.0 (366.5;525.5)
**TAP divided attention auditive (seconds)**								
1 month	579.5 (506.5;691.3)	635.0 (551.0;727.0)	512.0 (462.0;713.0)	593.0 (526.0;736.5)	566.5 (503.5;604.8)	591.0 (541.0;721.0)	604.0 (562.0;711.0)	672.5 (576.3;779.5) ***0.045**
6 months	602.0 (511.0;685.0)	617.5 (573.3;752.3)	482.0 (466.5;680.5)	579.0 (490.0;791.0)	545.5 (493.0;613.0)	603.5 (557.3;738.3)	646.5 (555.3;714.3)	654.0 (602.5;752.5)
12 months	603.0 (521.0;667.0)	630.0 (545.0 ;687.0)	521.0 (471.0;645.5)	591.0 (508.5;728.5)	560.0 (518.8;599.3)	601.5 (555.0;659.3)	648.5 (543.5;678.3)	663.0 (568.8;724.5)
24 months	539.0 (504.0;674.0)	649.0 (513.0;738.5)	623.0 (511.0;706.5)	500.5 (447.8;667.3)	515.0 (445.0;598.0)	577.5 (503.3;755.3)	606.0 (504.0;701.0)	673.0 (638.5;738.5) ***0.042**

**TAP divided attention visual (seconds)**								
1 month	789.5 (735.8;875.0)	796.0 (738.0;871.8)	735.0 (692.0;962.5)	786.0 (738.0;822.0)	782.5 (738.0;876.5)	743.5 (727.0;814.3)	802.0 (740.0;869.0)	860.0 (774.0;950.0) #**0.039**
6 months	760.0 (724.0;814.0)	834.0 (738.0;1000.0) ***0.016**	760.0 (696.0;980.5)	829.0 (741.0;858.5)	755.0 (738.3;795.3)	779.5 (696.8;1008.5)	788.0 (705.5;818.3)	857.0 (802.3;1020.5) ***0.002, †0.010**
12 months	761.5 (708.5;830.5)	817.5 (736.8;884.8)	780.0 (690.0;886.5)	799.0 (747.5;864.0)	753.0 (740.0;808.0)	781.0 (693.5;879.5)	767.0 (700.0;859.0)	834.0 (775.0;967.0)
24 months	799.0 (708.5;835.0)	791.0 (742.0;881.0)	802.0 (717.5;1002.5)	799.0 (704.5;925.8)	799.0 (725.0;867.0)	742.5 (652.5;873.3)	794.0 (707.0; 820.0)	798.0 (784.0;881.0)

Data are medians (Q1;Q3). *p-value vs total cohort of controls, †p-value vs corresponding matched controls, ‡p-value vs anterior thalamic stroke patients, #p-value vs paramedian thalamic stroke patients.
